# Nongenetic evolution of the tumor: from challenges to new therapeutic opportunities

**DOI:** 10.1002/1878-0261.13753

**Published:** 2024-10-18

**Authors:** Elisa Oricchio

**Affiliations:** ^1^ Swiss Institute for Experimental Cancer Research (ISREC), School of Life Sciences EPFL Lausanne Switzerland; ^2^ Swiss Cancer Center Leman (SCCL) Lausanne Switzerland

**Keywords:** chromatin, epigenetics, evolution, heterogeneity, transcription

## Abstract

The ability of cancer cells to change and adapt poses a critical challenge to identifying curative solutions. Tumor evolution has been extensively studied from a genetic perspective, to guide clinicians in selecting the most appropriate therapeutic option based on a patient's mutational profile. However, several studies reported that tumors can evolve toward more aggressive stages or become resistant to therapies without changing their genetic makeup. Indeed, several cell‐intrinsic and cell‐extrinsic mechanisms contribute to tumor evolution. In this viewpoint, I focus on how chromatin, epigenetic, and transcriptional changes contribute to tumor evolution, allowing cancer cells to transition to different cell states and bypass response to therapies. Although tumor nongenetic evolution is harder to trace and predict, understanding its principles might open new therapeutic opportunities.

## Graphical abstract

Tumors evolve to grow in different organs and to survive anticancer therapies. This involves the acquisition of genetic and epigenetic changes that allow cancer cells to adapt to different conditions. Understanding the mechanisms that drive tumor evolution is essential to prevent it and avoid its relapse after therapies.

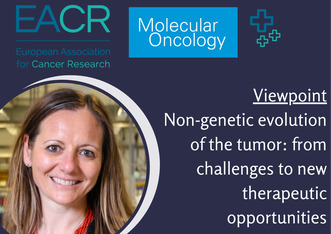

**Abbreviation**
3D: tridimensional

Cancer is a dynamic disease that continuously changes and evolves. The genetic evolution of a tumor is defined by the gradual accumulation of different genomic lesions. Tumor evolution has been extensively described through genomic sequencing of multiple regions of the same tumor sample and by collecting sequential samples at different stages of tumor development [1]. The accumulation and selection of new genetic mutations induce discrete and, mostly, nonreversible changes in the DNA sequence, representing bookmarks of the various steps of tumor evolution (Fig. 1). Hence, the combination of spatial and temporal analyses of genomic alterations detected in tumors has been used retrospectively to draw the path of tumor evolution [2, 3]. Treatment with anticancer therapies imposes new selective pressures on tumor cells. It facilitates the emergence of more aggressive and resistant clones through the acquisition of new genomic alterations or by selecting advantageous pre‐existing variants. However, tumors can also progress to a more aggressive stage or become resistant to therapies without the acquisition and/or selection of novel genetic lesions, highlighting the relevance of nongenetic paths of tumor evolution [4]. Epigenetic changes, chromatin remodeling, and transcriptional dynamics are some of the cell‐intrinsic processes that can contribute to the nongenetic evolution of a tumor (Fig. 1). Transcriptional and epigenetic heterogeneity of genetically identical cancer cells led to their classification in different cellular states [5]. During malignant progression or in response to therapies, cells can transition to different cell states defined by the acquisition of new epigenetic and transcriptional programs. The ability of cancer cells to shift between cell states depends on their capacity to remodel the chromatin, which can be facilitated by the acquisition of alterations in epigenetic regulators and the activation of transcription factors [6, 7]. Epigenetic remodeling influences and depends upon the tridimensional (3D) organization of the chromatin [8]. In the nucleus, the chromatin is compacted into distinct hierarchical structures called loops, chromatin domains, and compartments. Changes in histone post‐translation modifications can influence the conformation of chromatin loops connecting distant regulatory elements like enhancers and promoters and induce the repositioning of genomic regions in different compartments. The position of genomic regions in active, inactive, or intermediate compartments correlates with their epigenetic profile and gene expression [9, 10]. Interestingly, intermediate compartments have been associated with poised epigenetic states that can easily be switched on and off, while regions in active and inactive compartments are more difficult to change [11]. Intermediate compartment regions may be easily modulated in response to new environments, and this facilitates the acquisition of different epigenetic and transcriptional programs.

**Fig. 1:**
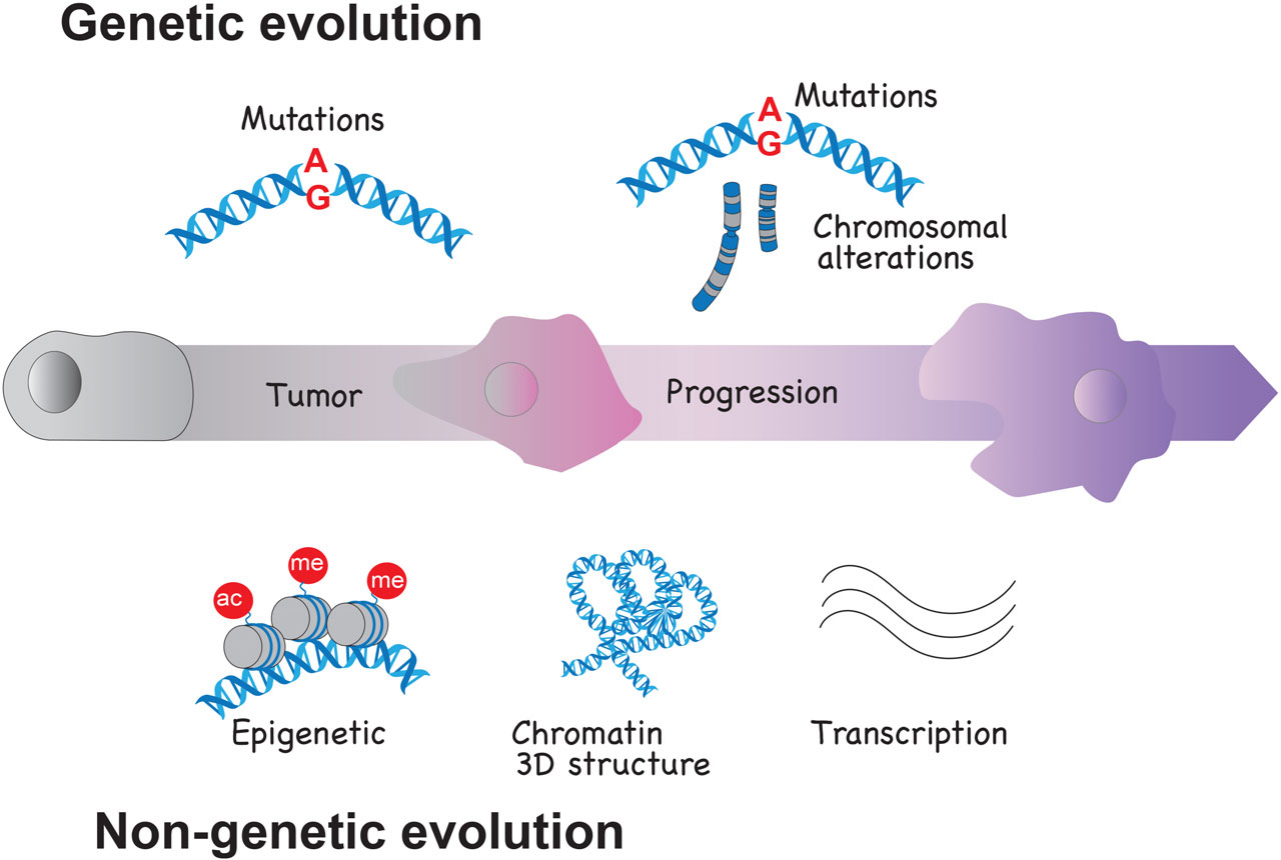
Parallel paths of genetic and not genetic evolution of the tumors are demarcated on one side by the accumulation of mutations and chromosomal changes, and on the other side by epigenetic, chromatin 3D structure, and transcriptional changes.

The interplay of multiple factors and the flexibility to modulate and reverse cell states makes tumor nongenetic evolution difficult to predict. Computational approaches to infer differentiation trajectories from transcriptional changes are challenged in this context because it is often not possible to establish the directionality of cell state changes. Experimentally, it is possible to introduce unique genetic barcodes in cancer cells, which allow to trace them over time. Thus, barcoding methods, in combination with single‐cell transcriptomic analyses, can be used to distinguish transcriptional evolutionary paths that emerge due to either cell adaptation or selection of pre‐existing populations during malignant progression or in response to therapies [12]. Some barcoding strategies have been designed to be able to extract the clones that will later lead to treatment resistance from the original cell population [13]. These techniques allow us to determine whether the transcriptional and epigenetic profiles of resistant cells were different from the rest of the cell population even before treatment or even if therapy resistance was stochastically acquired. However, some limitations remain in the application of these barcoding approaches, which rely on complex experimental settings that can influence the ability to retrieve all barcodes and, thus, rebuild the full evolutionary trajectories of resistant clones. Moreover, it is not possible to use these approaches in primary patient tumor samples. For this, it might be interesting to identify cell‐intrinsic barcodes such as, for example, patterns of DNA methylation that could be used to trace cancer cells without additional genetic manipulation [14, 15].

Cancer cell plasticity from the therapeutic perspective can represent a double‐edged sword. On the one hand, the reversibility of the process suggests that cancer cells can be epigenetically reprogrammed. Since several inhibitors have been developed to control the activity of epigenetic modulators, it can be envisioned that these inhibitors could be used to steer tumor evolution, pushing and maintaining cancer cells in a therapeutically controllable cellular state. On the other hand, transcriptional and epigenetic plasticity may lead to the emergence and selection of unexpected states to bypass treatment efficacy once again. Treatment with chemotherapy or targeted therapies often results in cycles of response and relapse, which has been associated with the presence of persister tumor cells in primary tumors. These cells represent a small slow‐growing cell population with high transcriptional plasticity, and they are responsible for tumor relapse even after several years. Persistent cells can pre‐exist in tumors by assuming a dormant state, making them resistant to therapies, but during the remission phase, they can reactivate proliferative gene expression programs. Therapeutic strategies to steer proliferating tumor cells into a dormant state or to reprogram the cell toward a less malignant cell state could represent possibilities to exploit cellular plasticity to prevent tumor relapse.

Finally, we cannot separate tumor epigenetic and genetic evolution, as the two processes influence each other. The ability of genetic mutations to elicit their oncogenic role depends on the transcriptional and epigenetic status of the cells. Mutations in oncogenes and tumor suppressors can be detected in healthy tissues [16], but they only assume oncogenic competence when expressed in a specific cellular context [17]. Similarly, whole genome doubling can be detected in healthy liver or precancerous esophageal cells. However, whole genome doubling combined with the loss of p53 promotes chromosomal instability and initiates the remodeling of chromatin 3D structure, resulting in the accumulation of chromosomal alterations and local epigenetic modifications promoting oncogene expression. In this context, chromatin modifications and genomic instability create a cellular context that facilitates oncogenic transformation [8].

In addition to multiple cell‐intrinsic mechanisms, the tumor microenvironment, microbiome, nutrient availability, and environmental stress can influence cancer development and response to therapy. These mechanisms co‐exist and synergize within a tumor. It will be important to develop predictive models accounting for multiple evolutionary mechanisms and their interactions to anticipate treatment responses and design the most effective therapeutic protocols. As there is not one unique way for a tumor to evolve, there is not one unique way to cure it.

## Conflict of interest

The author declares no conflict of interest.
